# Best compromise nutritional menus for childhood obesity

**DOI:** 10.1371/journal.pone.0216516

**Published:** 2020-01-24

**Authors:** Paul Bello, Pedro Gallardo, Lorena Pradenas, Jacques A. Ferland, Victor Parada

**Affiliations:** 1 Departamento de Ingeniería Industrial, Universidad de Concepción, Concepción, Chile; 2 Département d’Informatique et Recherche Opérationnelle, Université de Montréal, Montréal, Canada; 3 Departamento de Ingeniería Informática, Universidad de Santiago de Chile, Santiago, Chile; Universidad Nacional Autonoma de Mexico Instituto de Investigaciones en Ecosistemas y Sustentabilidad, MEXICO

## Abstract

Childhood obesity is an undeniable reality that has rapidly increased in many countries. Obesity at an early age not only increases the risks of chronic diseases but also produces a problem for the whole healthcare system. One way to alleviate this problem is to provide each patient with an appropriate menu that is defined by a mathematical model. Existing mathematical models only partially address the objective and constraints of childhood obesity; therefore, the solutions provided are insufficient for health specialists to prepare nutritional menus for individual patients. This manuscript proposes a multiobjective mathematical programming model to aid in healthy nutritional menu planning that may prevent childhood obesity. This model provides a plan for combinations and amounts of food across different schedules and daily meals. This approach minimizes the major risk factors of childhood obesity (i.e., glycemic load and cholesterol intake). In addition, this approach considers the minimization of nutritional mismatch and total cost. The model is solved using a deterministic method and two metaheuristic methods. Test instances associated with children aged 4–18 years were created with the support of health professionals to complete this numerical study. The quality of the solutions generated using the three methods was similar, but the metaheuristic methods provided solutions in a shorter computational time. These results are submitted to statistical hypothesis tests to be validated. The numerical results indicate proper guidelines for personalized plans for individual children.

## Introduction

Childhood obesity has shown rapid growth in many countries, but this growth may be partially mitigated through the use of optimization mathematical models. This noncommunicable disease is a major public health concern because a child who is obese at an early age displays increased risks of cardiovascular, pulmonary, metabolic, gastrointestinal, skeletal, psychological and other diseases in adulthood [1][2][3]. Additionally, the evidence reveals a positive correlation between obesity/being overweight in childhood and these conditions in adulthood [4]. Therefore, interventions administered during childhood have great potential because healthy eating habits can be developed at this stage [5]. As a method to address this problem, a health professional must specify the combination and amount of food that the patient should consume at different meal times during the day to ensure the appropriate intake of the nutrients of interest during the planning period. These facts introduce a particular type of operational research problem called the Nutritional-Menu Planning Problem (NMPP). The NMPP is an NP-Hard problem [6], and in practical terms, the method usually used to solve it consists of manually constructing menus through a trial-and-error process that is extremely inefficient and does not guarantee an appropriate menu for each patient.

NMPP variants approached using mathematical models have different objective functions. Stigler [7] and Dantzig [8] were the first to propose the goal of minimizing the total cost of the diet problem. Bas [9] studied the minimization of a risk factor for patients with a high glycemic load and metabolic diseases. Orešković, Kljusurić, and Šatalić [10] maximized the palatability of a menu based on patient preferences by assigning a weight to the objective function in the specific case of vegetarian menus. Masset et al. [11] and Okubo et al. [12] minimized the difference between the quantities currently ingested and the recommended amount while satisfying nutritional requirements. Complementary diets for 6- to 24-month-olds [13] and the planning of nutritional menus at a school in Southeast Asia for 13- to 18-year-olds were studied by considering total cost minimization [14]. In some situations, cost minimization alone is insufficient to obtain the proper diet. Other objectives are also relevant, leading to the multiobjective NMPP that we denote as MO-NMPP.

Several MO-NMPP studies have been conducted. A multiobjective model more completely represents the real problem addressed by the NMPP. Koroušić [6] [15] addressed both economic and aesthetic aspects when generating food menus. The multiobjective model optimizes cost, functionality, seasonality, and other aspects, such as flavor, consistency, color, temperature, shape and method of preparation. Donati et al. [16] presented a multiobjective model to generate diets at the lowest cost while minimizing the environmental effect of its production, which was measured as equivalent carbon dioxide emissions and land and water use. Van Mierlo, Rohmer and Gerdessen [17] studied a similar situation that minimized fossil fuel depletion instead of cost minimization. The authors found that the existing models for MO-NMPP are focused on general issues that are valid for an obese individual. However, childhood obesity treatments must consider the child’s development. Thus, noticeably restrictive diets in terms of calories are not recommended because children are developing. Additionally, the recommended menu must encourage the development of healthy eating habits while minimizing exposure to risk factors such as energy-dense, high-fat, high-sugar and high-salt foods. Furthermore, an appropriate glycemic load and an average daily cholesterol intake are necessary. Moreover, the minimum nutritional mismatch between the nutritional contributions provided by the menu and the amount recommended by specialized organizations is an essential condition that the best compromise solution must satisfy. By including all of these components in the original multiobjective problem, we introduce the Multiobjective Nutritional-Menu Planning Problem for Childhood Obesity (MO-NMPP-CHO).

This paper proposes an approach for the MO-NMPP-CHO that considers the minimization of the main risk factors for the development of chronic childhood obesity. The concept of nutritional mismatch is considered, which slightly relaxes the constraints. Moreover, the classic objective of minimizing the average daily cost of the menu was considered to avoid limiting the applicability of the menus to sectors with lower incomes, which adds to the nutritional constraints suggested by specialized organizations. With the help of a specialist, we created a set of numerical instances that were solved using a deterministic method and two metaheuristic methods.

The remainder of this paper is organized as described below. Section 2 describes the methods used to complete our analysis. In Section 2.1, we introduce the multiobjective mathematical programming model (MO-NMPP-CHO) that we proposed to control and prevent childhood obesity. Then, we propose two solution strategies to complete the analysis in Section 2.2: an approach based on the ℇ-constraint method and two other evolutionary approaches. Section 2.3 describes tools related to problem generation, the performance measures of the solution strategies, and their statistical analysis. The numerical experimentation and the discussion of the results are summarized in Sections 3 and 4, respectively. Finally, Section 5 presents the main conclusions of this study.

## Methods

In this section, we introduce the multiobjective model for the MO-NMPP-CHO, the approaches used to address it, and the analysis tools used to complete the analysis.

### A multiobjective approach for the MO-NMPP-CHO

This section presents the model for the MO-NMPP-CHO. The proposed approach minimizes the main risk factors for the development of the chronic diseases associated with childhood obesity, nutritional mismatch and the average daily cost of the generated menus. **The definitions of the parameters and variables included in the model are summarized as follow:**

Description of sets and subindexes:

*A*: Number of fatty acids considered, *a* = 1,…, *A*

*G*: Number of food groups considered, *g* = 1,…, *G*

*I* (*k*, *j*): Number of meals that can be served of dish *j* during mealtime *k*, *i* = 1, …, *I* (*k*, *j*)

*J* (*k*): Number of dishes to be served during mealtime *k*, *j* = 1,…, *J* (*k*)

*K*: Number of mealtimes considered, *k* = 1,…, *K*

*L*: Number of days considered for menu planning, *l* = 1,…, *L*

*M*: Number of macronutrients considered, *m* = 1,…, *M*

*V*: Number of vitamins considered, *v* = 1,…, *V*

*H*: Number of minerals considered, *h* = 1,…, *H*

Description of Parameters:

*C*_*kji*_: Cost of food *i* in dish *j* at mealtime *k*

*CG*_*kji*_: Units of the estimated glycemic load by food portion *i* of dish *j* at mealtime *k*

*AMN*_*kjim*_: Grams of macronutrient *m* by a portion of food *i* of dish *j* at mealtime *k*

*EM*_*m*_: Kilojoules contributed by one gram of macronutrient *m*

*PEMS*_*m*_/*PEMI*_*m*_: Maximum/minimum fraction of energy contributed by macronutrient *m*

*AML*_*kjia*_: Fatty acid *a* contributed by a portion of food *i* of dish *j* at mealtime *k*

*EA*_*kji*_: Totals of kilojoules contributed by a portion of food *i* of dish *j* at mealtime *k*

*ED*: Total kilojoules required each day

*ECS*_*k*_/*ECI*_*k*_: Maximum/minimum fraction of daily energy provided at mealtime *k*

*RL*_*a*_: Maximum fraction of energy contributed by the fatty acid *a*

*AV*_*kjiv*_: Vitamin *v* intake by a portion of food *i* of dish *j* at mealtime *k*

*RSV*_*v*_/*RIV*_*v*_: Maximum/minimum intake of vitamin *v* each day

*AM*_*kjih*_: Contribution of mineral *h* from a portion of food *i* of dish *j* at mealtime *k*

*RSM*_*h*_/*RIM*_*h*_: Maximum/minimum consumption of mineral *h* each day

*AF*_*kji*_: Contribution, in grams, of dietary fiber from a portion of food *i* of dish *j* at mealtime *k*

*RF*/*FD*: Maximum/minimum number of grams of dietary fiber recommended each day

*Gr*_*kjig*_: Indicates if food *i* in dish *j* at time *k* belongs to group *g*

*RGDS*_*g*_/*RGDI*_*g*_: Maximum/minimum number of daily dishes of group *g* recommended for good nutrition

*RGSS*_*g*_/*RGSI*_*g*_: Maximum/minimum number of dishes per week of group *g* recommended

*LSP*/*LIP*: Minimum/maximum number of portions allowed

Description of Variables:

*Rv*_*lv*_: Deviation in the amount of vitamin *v* in relation to the recommended amount on day *l*

*Rmi*_*lh*_: Deviation in the amount of mineral *m* in relation to the recommended amount on day *l*

*Rf*_*l*_: Deviation in the amount of dietary fiber in relation to the recommended amount on day *l*

*Ra*_*la*_: Deviation in the energy level provided by fatty acid *a* on day *l*

*Re*_*l*_: Deviation in the total energy on day *l*

*Rhc*_*lk*_: Deviation in the energy level provided at mealtime *k* on day *l*

*Rma*_*lm*_: Deviation in the energy level provided by macronutrient *m* on day *l*

*Rgd*_*lg*_: Deviation in the level of food group *g* consumed on day *l*

*Rgs*_*g*_: Deviation in the level of food group *g* consumed in one week.

*y*_*kjil*_: 1, if food *i* is in dish *j* at time *k* on day *l* and 0 otherwise

*x*_*kjil*_: Amount of portions of food *i* served in dish *j* at time *k* on day *l*

The model that enables the generation of food plans for children to reduce the risk of childhood obesity is presented in Eqs (1)–(19).

$$Min\;Z1=\frac{\left(\sum_{l=1}^{L}\sum_{k=1}^{K}\sum_{j=1}^{J\left(k\right)}\sum_{i=1}^{I\left(j,k\right)}C_{kji}*x_{kjil}\right)}{L}$$ (1)

$$Min\;Z2=\frac{\left(\sum_{l=1}^{L}\sum_{k=1}^{K}\sum_{j=1}^{J\left(k\right)}\sum_{i=1}^{I\left(j,k\right)}x_{kjil}*AML_{kji3}\right)}{L}$$ (2)

$$Min\;Z3=\frac{\left(\sum_{l=1}^{L}\sum_{k=1}^{K}\sum_{j=1}^{J\left(k\right)}\sum_{i=1}^{I\left(j,k\right)}CG_{kji}*x_{kjil}\right)}{L}$$ (3)

$$Min\;Z4=\left(\overset{L}{\underset{l=1}{\sum }}\left(\overset{V}{\underset{v=1}{\sum }}Rv_{lv}+\overset{H}{\underset{h=1}{\sum }}Rmi_{lh}+\overset{A}{\underset{a=1}{\sum }}Ra_{la}+\overset{K}{\underset{k=1}{\sum }}Rhc_{lk}+\overset{M}{\underset{m=1}{\sum }}Rma_{lm}+\overset{G}{\underset{g=1}{\sum }}Rgd_{\mathrm{lg}}+Rf_{l}+Re_{l}\right)+\overset{G}{\underset{g=1}{\sum }}Rgs_{g}\right)/\left(L*9\right)$$ (4)

Subject to:

$$ED*\left(1-Re_{l}\right)\leq \overset{K}{\underset{k=1}{\sum }}\overset{J\left(k\right)}{\underset{j=1}{\sum }}\overset{I\left(j,k\right)}{\underset{i=1}{\sum }}x_{kjil}*EA_{kji}\leq ED*\left(1+Re_{l}\right)\;\forall l$$ (5)

$$PEMI_{m}*ED*\left(1-Rma_{lm}\right)\leq \overset{K}{\underset{k=1}{\sum }}\overset{J\left(k\right)}{\underset{j=1}{\sum }}\overset{I\left(j,k\right)}{\underset{i=1}{\sum }}x_{kjil}*EM_{m}*AMN_{kjim}\leq PEMS_{m}*ED*\left(1+Rma_{lm}\right)\;\forall l,m$$ (6)

$$ECI_{k}*ED*\left(1-Rhc_{lk}\right)\leq \overset{J\left(k\right)}{\underset{j=1}{\sum }}\overset{I\left(j,k\right)}{\underset{i=1}{\sum }}x_{kjil}*EA_{kji}\leq ECS_{k}*ED*\left(1+Rhc_{lk}\right)\;\forall k,l$$ (7)

$$\overset{K}{\underset{k=1}{\sum }}\overset{J\left(k\right)}{\underset{j=1}{\sum }}\overset{I\left(j,k\right)}{\underset{i=1}{\sum }}x_{kjil}*EM_{3}*AML_{kjia}\leq RL_{a}*ED*\left(1+Ra_{la}\right)\;\forall a\neq 3,l$$ (8)

$$RIV_{v}*\left(1-Rv_{lv}\right)\leq \overset{K}{\underset{k=1}{\sum }}\overset{J\left(k\right)}{\underset{j=1}{\sum }}\overset{I\left(j,k\right)}{\underset{i=1}{\sum }}x_{kjil}*AV_{kjiv}\leq RSV_{v}*\left(1+Rv_{lv}\right)\;\forall v,l$$ (9)

$$RIM_{h}*\left(1-Rmi_{lh}\right)\leq \overset{K}{\underset{k=1}{\sum }}\overset{J\left(k\right)}{\underset{j=1}{\sum }}\overset{I\left(j,k\right)}{\underset{i=1}{\sum }}x_{kjil}*AM_{kjih}\leq RSM_{h}*\left(1+Rmi_{lh}\right)\;\forall h,l$$ (10)

$$FD\left(1-Rf_{l}\right)\leq \overset{K}{\underset{k=1}{\sum }}\overset{J\left(k\right)}{\underset{j=1}{\sum }}\overset{I\left(j,k\right)}{\underset{i=1}{\sum }}x_{kjil}*AF_{kji}\leq RF*(1+Rf_{l})\;\forall l$$ (11)

$$RGDI_{g}*\left(1-Rgd_{lg}\right)\leq \overset{K}{\underset{k=1}{\sum }}\overset{J\left(k\right)}{\underset{j=1}{\sum }}\overset{I\left(j,k\right)}{\underset{i=1}{\sum }}y_{kjil}*Gr_{kjig}\leq RGDS_{g}*\left(1+Rgd_{lg}\right)\;\forall l,g$$ (12)

$$RGSI_{g}\left(1-Rgs_{g}\right)\leq \overset{L}{\underset{l=1}{\sum }}\overset{K}{\underset{k=1}{\sum }}\overset{J\left(k\right)}{\underset{j=1}{\sum }}\overset{I\left(j,k\right)}{\underset{i=1}{\sum }}y_{kjil}*Gr_{kjig}\leq RGSS_{g}*\left(1+Rgs_{g}\right)\;\forall g$$ (13)

$$\overset{I\left(j,k\right)}{\underset{i=1}{\sum }}y_{kjil}=1\;\forall j,k,l$$ (14)

$$y_{32il}+y_{32i\left(l+1\right)}\leq 1\;\forall i,l$$ (15)

$$y_{62il}+y_{62i\left(l+1\right)}\leq 1\;\forall i,l$$ (16)

$$LIP*y_{kjil}\leq x_{kjil}\leq LSP*y_{kjil}\;\forall i,j,k,l$$ (17)

$$x_{kjil},Rv_{lv},Rmi_{lh},Rf_{l},Ra_{la},Re_{l},Rhc_{lk},Rma_{lm},Rgd_{lg},Rgs_{g}\geq 0\;\forall i,j,k,l,v,h,a,m,g$$ (18)

$$y_{kjil}\in \left\{0,1\right\}\;\forall i,j,k,l$$ (19)

The first four Eqs (1) to (4), correspond to the objective functions. The first objective function (1) minimizes the average daily cost of the food plan [7]. The second objective function (2) minimizes the average daily cholesterol intake to reduce the negative effects of fat consumption. The third objective function (3), which was proposed by Bas [9], minimizes the average daily glycemic load of the menu. The glycemic load (*GL*) corresponds to the glycemic index (*GI*), which is adjusted by a specific amount of carbohydrates (*GL* = carbohydrates x *GI*/100). This concept is a topic of interest because the consumption of foods with a low glycemic index reduces the risk of diseases associated with hyperinsulinemia (excess insulin in the blood), such as diabetes mellitus and cardiovascular diseases, while also decreasing the sensation of hunger [18]. Finally, the fourth objective (4) minimizes the average daily nutritional mismatch of the generated menu, whose elements are specified in constraints (5)–(13).

Constraints (5), (6), (7) and (8) limit the total daily energy input in kilojoules contributed by each group of macronutrients each day, the energy contribution of different meals schedules, and energy contributions of saturated and unsaturated fatty acids, respectively. Constraints (9) and (10) ensure that the requirements for vitamins and minerals were satisfied in this study according to the recommended and tolerable levels of intake, as specified by specialized organizations. In addition, other elements must be provided, although they are not considered as nutrients. Thus, constraint (11) controls the daily consumption of dietary fiber. Constraints (12) and (13) ensure the proper daily and weekly intake of different food groups, as suggested by experts. Constraints (14), (15), (16) and (17) specify the appropriate menus. Thus, constraint (14) requires that all dishes served at different meal times on different days have an assigned food. Constraints (15) and (16) ensure that no main dish is served during two consecutive lunches or two consecutive dinners, respectively. Constraint (17) limits the size of portions that can be assigned.

Finally, constraints (18) and (19) define the types of variables in the model. The first variables were the assigned portion and mismatch levels, which must be greater than or equal to zero. The second set includes binary variables associated with the decision regarding whether to consider food under the established conditions. Then, the resulting model is a mixed integer linear programming problem.

### Solution strategies

Unlike optimization problems with only one objective function, in the multiobjective case, a set of nondominated (efficient) solutions is sought instead of an optimal solution. For example, if a multiobjective model includes several minimization objectives *Z*_*i*_ (*x*), then a solution *y* dominates solution *x* if *Z*_*i*_ (*y*) ≤ *Z*_*i*_ (*x*) for every objective *i*, and at least one objective *i* exists such that *Z*_*i*_ (*y*) < *Z*_*i*_ (*x*). The set of solutions that are not dominated by another solution in the objective space is known as the Pareto border [19]. The model for MO-NMPP-CHO is solved using three different methods. The Ɛ-constraint method [20] is implemented using the General Algebraic Modeling System (GAMS/CPLEX solver) [21]; two multiobjective evolutionary algorithms (MOEA) are implemented in C++: Nondominated Sorting Genetic Algorithm II [22], which is also known as NSGA-II, and Strength Pareto Evolutionary Algorithm 2 [23], which is also known as SPEA2. A set of test instances associated with boys and girls aged 4–18 years was created with the support of health professionals to complete the numerical study.

#### An approach for the MO-NMPP-CHO based on the Ɛ-constraint method

The purpose of the Ɛ-constraint method is to transform a multiobjective problem into several mono-objective problems to optimize one objective function, whereas those problems that become part of the constraints are limited by values ε. For example, let us consider the multiobjective model specified by Eqs (20) and (21), where objective *Z* is a vector of *p* functions *Z*_*i*_ (*i* = 1,…, *p*) and *F*_*d*_ is the feasible region. The Ɛ-constraint method generates several mono-objective models, as illustrated in Eqs (22)–(24).

$$Min\;Z\left(x_{1},x_{2},\ldots ,x_{n}\right)=\left[Z_{1}\left(x_{1},x_{2},\ldots ,x_{n}\right),Z_{2}\left(x_{1},x_{2},\ldots ,x_{n}\right),\ldots ,Z_{p}\left(x_{1},x_{2},\ldots ,x_{n}\right)\right]$$ (20)

Subject to,

$$\left(x_{1},x_{2},\ldots ,x_{n}\right)\in F_{d}$$ (21)

$$Min\;Z\left(x_{1},\ldots ,x_{n}\right)=Z_{i}\left(x_{1},\ldots ,x_{n}\right)$$ (22)

Subject to,

$$\left(x_{1},x_{2},\ldots ,x_{n}\right)\in F_{d}$$ (23)

$$Z_{k}\left(x_{1},\ldots ,x_{n}\right)\leq \varepsilon_{k}\;k=\left\{1,\ldots ,p\right\},k\neq i$$ (24)

Our model includes *p* = 4 objective functions, and *F*_*d*_ is specified by constraints (5)-(19). The described process is applied to each of the four objectives. The basic issue is to determine the appropriate values of ε_*i*_ (*i* = 1,…,4). Thus, a separate problem, as illustrated in Eqs (25) and (26), is solved for each objective function *Z*_*i*_, and the optimal solution $\left({\bar{x}}^{i},{\bar{y}}^{i}\right)$ is used to specify the vector $\left[Z_{1}({\bar{x}}^{i},{\bar{y}}^{i}),\ldots ,Z_{4}({\bar{x}}^{i},{\bar{y}}^{i})\right]$. Then, the range of values $\left[\min_{i\in \{1,\ldots ,4\}}Z_{p}({\bar{x}}^{i},{\bar{y}}^{i}),\max_{i\in \{1,\ldots ,4\}}Z_{p}({\bar{x}}^{i},{\bar{y}}^{i})\right]$ for each *ε*_*p*_ (*p* = 1,…, 4) is divided into *t* parts to determine (*t*+1) values for *ε*_*p*_. In our case, *t* = 2 generates 3 different values for *ε*_*p*_.

$$Min\;Z\left(x,y\right)=Z_{i}\left(x,y\right)$$ (25)

Subject to,

$$\left(x,y\right)\in F_{d}$$ (26)

For each objective *Z*_*i*_ (*i* = 1,…,4), the mono-objective model in Eqs (22)–(24) is solved for each combination of different values of *ε*_*k*_, *k* ϵ {1,…,4}, where *k* ≠ *i* in their sets of values (i.e., 27 different problems are solved for each *i*), to complete the Ɛ-constraint method. The models generated for different *ε* combinations are solved using the GAMS/CPLEX solver with the Branch-and-Cut algorithm. After the solutions for all of the models generated by the combination of *ε* values are obtained, the nondominance in the objective space is used for all solutions, which generates the Pareto border approximation.

#### Two evolutionary approaches for the MO-NMPP-CHO

In an evolutionary approach, a complete population of solutions is modified during the process. Among these methods, a subclassification known as evolutionary algorithms presents multiple advantages to address multiobjective problems [24]. In fact, evolutionary algorithms are characterized by imitating the evolutionary process of the species regarding the survival of the fittest, i.e., a population of individuals (solutions to the problem) is modified after several generations through the application of parent selection rules, crossover strategies and mutation strategies. Thus, the following series of elements must be introduced to proceed:

- Encoding the solution: definition of the coded representation (or chromosome) of individuals in the population in both the objective space and the decision space.
- Fitness assignment: definition of a strategy to assign a value to each individual to motivate its aptitude to be part of the next generation.
- Mating selection: definition of the strategy to select individuals to be parents of new solutions.
- Environmental selection: definition of the strategy to decide the members of the current population that will be included in the population of the next generation.
- Reproduction strategy: definition of the mutation and crossover operators to generate the next generation with the probability of applying each operator.
- Initialization of population: definition of the population size and strategy to create the initial population.
- Stop criterion: definition of a criterion that enables the algorithm to stop the calculation after fulfilling a condition.

We consider two evolutionary algorithms, NSGA-II and SPEA2, to address the MO-NMPP-CHO. First, we defined the identical operators and strategies to implement both methods; then, we specified the different operators and particular strategies in each method.

**Fig 1:**
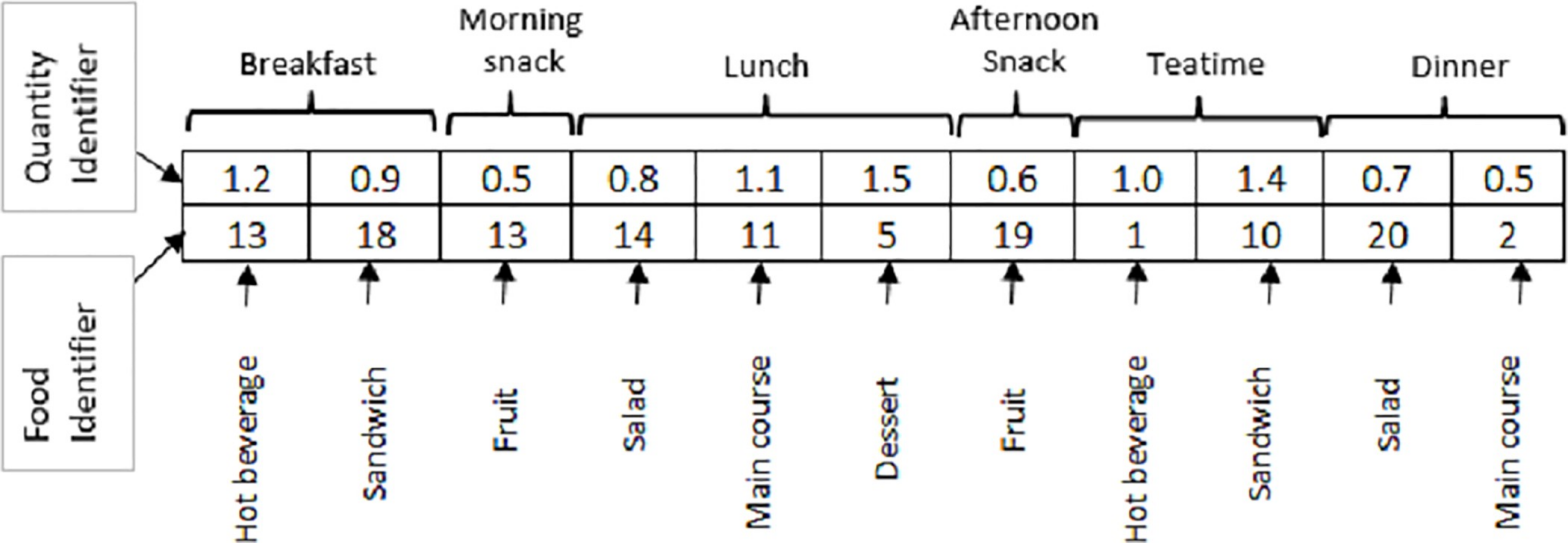
Representation of an individual in the decision space, planning for one day.

*Encoding the solution*: A solution in the decision space is represented using two rows and *T* columns, where *T* is the number of days multiplied by the number of dishes that should be served per day. Fig 1 illustrates the attributes of each row and column to encode the solution in the decision space. The first row includes the number of food portions to be served, and the second row includes an identifier of the food to be served. The position of each column considers different characteristics (e.g., the meal time to which it belongs and the dish in the meal). The representation of an individual in the objective space corresponds to a vector whose size is equal to the number of objectives (4 in this case).

*Reproduction strategy*: For both metaheuristic methods, we used a crossover operator with *M* crossing points [19], where *M* is equal to the number of days to be planned. The mutation operator modifies the number of points equal to the number of days to be planned and thus both the food and amount of food portion are randomly reallocated. Two different children are generated when the crossover operator is applied, and one child is randomly selected. A strategy of crossover-OR-mutation is used so that at least one operator (crossover or mutation) is applied during the crossbreeding application [25].

*Initial population*: Individuals are randomly created to generate the initial population and ensure diversity within the objective space. However, they become members of the population if they are not clones of any of the existing individuals in the initial population.

*Stop criterion*: The termination condition for both metaheuristic methods is the fulfillment of *G*_max_ generations or a maximum running time of 1,800 seconds.

**Fig 2:**
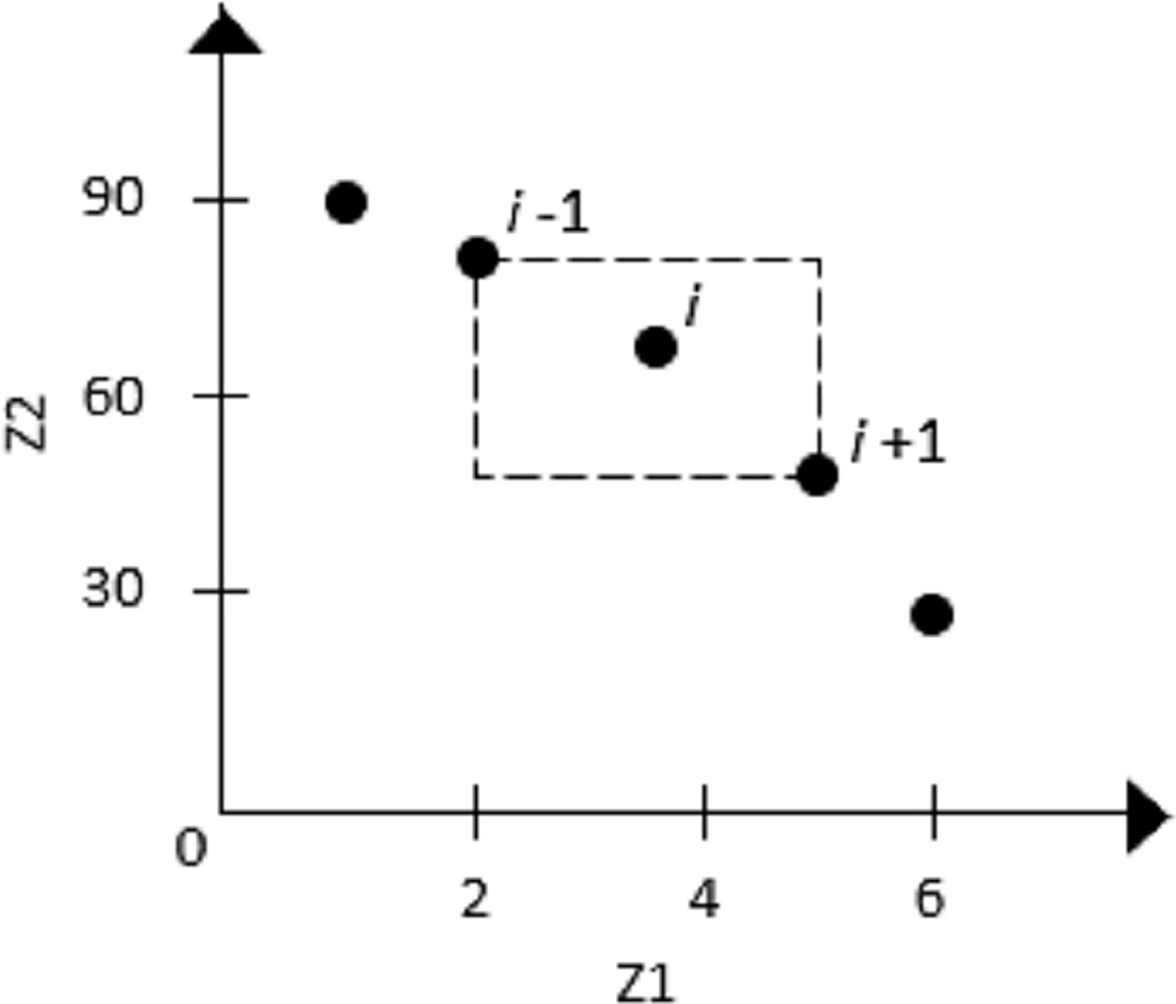
Example of crowding distance. Each point is a nondominated solution.

The elements listed below must be specified to implement NSGA-II for the MO-NMPP-CHO. First, the fitness allocation is based on the dominance depth criterion that generates several layers in the population; namely, a population of individuals creates a better-quality layer (i.e., ranking 1), which includes individuals who are not dominated by others. The second layer with ranking 2 includes the individuals who are not dominated by others in the remaining population. The same principle applies to create other layers with higher rankings. The second element that specifies fitness is the density estimator, which is designated the crowding distance and consists of estimating the perimeter of the cuboid formed by the neighbors closest to the individual in the objective space illustrated in Fig 2 for a bi-objective maximization problem. Thus, the operator of a crowding comparison specifies that an individual dominates another if it has a better ranking or equal ranking with a greater crowding distance.

The parents who create the new population through the application of genetic operators are selected from the current population through a binary tournament using the crowding comparison operator to specify the fittest individuals. Environmental selection is performed by adding the best layers from the current population to the new population until it reaches its preset size. If the population preset size is unable to be achieved exactly, then individuals with better density indicators are added from the last candidate layer for inclusion until the size of the new population is attained. At the end of the procedure, the individuals with a ranking of 1 correspond to nondominant individuals and form the Pareto border approximation. We include the strategy of eliminating the overlapping solutions in the objective space after creating the new population, as described in a previous study [26]. Hence, in the worst case, *N* individuals are present instead of clones to continue the process because the initial population does not contain clones. A parameterization procedure was conducted to tune the parameters of NSGA-II using a 2^*k*^ factorial design [27] with a confidence level of 95% that resulted in the following parameters: population size, 300; the maximum number of generations, 500; and the probability of applying the crossover operator instead of the mutation operator, 95%.

SPEA2 method for the MO-NMPP-CHO is characterized as described below. Unlike NSGA-II, SPEA2 ensures elitism through an external file in addition to the main population of individuals. The size of the external file remains fixed because of the truncation operator. Hence, when the sample exceeds the permitted size for the external file, individuals with a smaller distance to another individual in the objective space are iteratively eliminated until the sample has attained the permitted size, thereby avoiding the elimination of boundary solutions. The density estimator corresponds to the inverse of the Euclidean distance in the objective space between the individual and the *k*-th closest individual, where *k* is equal to the integer part of the square root of the sum of the size of the main population and the size of the external file. The strength value for each individual must be calculated to obtain the fitness of an individual. Therefore, the fitness of an individual is equal to the sum of its raw fitness that corresponds to the sum of the strength value of the individuals who dominates it and its density estimator.

The environmental selection process used to generate the external file of the next generation was applied by copying the nondominant individuals of the current main population and the external file into the external file of the next generation. The environmental selection process employed to generate the main population of the next generation was performed by applying genetic operators to the parents selected from the external file of the next generation. The parents were selected through a binary tournament using their fitness. When the stop criterion was fulfilled, the individuals in the external file with a fitness value less than one corresponded to the nondominant individuals who formed the Pareto border approximation.

The parameterization for SPEA2 was performed using a 2^*k*^ factorial design with a 95% confidence level that resulted in the following parameters: size of the main population, 300; maximum number of generations, 500; probability of applying the crossover operator instead of the mutation operator, 80%; and external file size: 50% of the main population size.

#### Implementation of solution strategies

The proposed mathematical programming model was solved with the three methods described above. For the Ɛ-constraint method, each problem instance was solved using GAMS software, version 24.3.3 with IBM ILOG CPLEX *Optimization Studio* solver, version 12.06.1 [21]. The metaheuristics were implemented in C++. All implementations were solved using a computer with an i7 Intel Core processor operating at 2.40 GHz and 8 GB of RAM.

### Analysis tools

Different elements are introduced to complete the analysis: problem instances, performance measures of the strategies, and their statistical analysis.

#### Problems generated

Because no instance was available in the literature to complete our analysis, six problems were built with the support of two health professionals from two public clinical centers. Both professionals have been working for several years in the diagnosis and treatment of childhood obesity. The problem instances were constructed based on the WHO nutritional recommendations and the experience of health professionals after considering the needs of individual children diagnosed with obesity in different age groups.

The food composition database used in the present study corresponds to the database available in the INTA (Institute of Nutrition and Food Technology) of the University of Chile (https://inta.cl/tabla-de-composicion-de-alimentos-2018).

#### Model testing

Different indicators **(**due to Talbi [17]) are used to complete the analysis of the results generated with the methods. The notation || · || indicates the Euclidian distance in the objective space, and | · | indicates the cardinality of a set. The *Extent* indicator *I*_*ex*_(*A*) (27) is used to analyze the diversity within population *A* generated with a particular method, where *n* is the number of objective functions, *Z*_*i*_ (*u*) is the value of the *i*-th objective function, and *Z*(*u*) is the vector of the objective functions of individual *u*.

$$I_{ex}\left(A\right)={\left(\overset{n}{\underset{i=1}{\sum }}\left(max_{u,v\in A}\|Z_{i}\left(u\right)-Z_{i}\left(v\right)\|\right)\right)}^{1/2}$$ (27)

The *Generational Distance I*_*GD*_(*A*,*R*) (28) measures the distance between the final population *A* and the initial population *R*, thus measuring the improvement obtained with heuristic methods. The method with the best performance in calculating this indicator achieves the greatest distance between its init**ial and final population**s.

$$I_{GD}\left(A,R\right)={\left(\underset{u\in A}{\sum }min_{v\in R}\|Z\left(u\right)-Z{(v)\|}^{2}\right)}^{1/2}/\left|R\right|$$ (28)

The *Contribution* indicator *Cont*(*PF*_1_/*PF*_2_) (29) is used to measure the contribution of the nondominated solutions of two methods using approximations of their Pareto fronts *PF*_1_ and *PF*_2_. When combining the solutions from these methods, *PF* denotes the intersection of sets *PF*_1_ and *PF*_2_, PF* includes the nondominated solutions in *PF*_1_ ∪ *PF*_2_, *W*_*1*_ is the set of solutions of *PF*_1_ that dominates a solution in *PF*_2_, and *N*_*1*_ corresponds to the set of solutions of *PF*_1_ that do not interact with the solutions of *PF*_2_ (i.e., solutions in *N*_*1*_ that do not dominate any solution, are not dominated by any solution and are not clones of any solution of *PF*_2_). Finally, *Cont* (*PF*_1_/*PF*_2_) computes the proportion of nondominated solutions that *PF*_1_ allocates to PF*, *Cont* (*PF*_2_/*PF*_1_) computes the proportion of nondominated solutions that *PF*_2_ allocates to PF*, and *Cont* (*PF*_1_/*PF*_2_) + *Cont* (*PF*_2_/*PF*_1_) = 1. For example, if *Cont* (*PF*_1_/*PF*_2_) is greater than 0.5, then *Cont* (*PF*_2_/*PF*_1_) is less than 0.5; the method that generates *PF*_1_ is better than the method that generates *PF*_2_ in terms of convergence to the Pareto frontier.

$$Cont\;\left(PF_{1}/PF_{2}\right)=\left(\frac{\left|PF\right|}{2}+\left|W_{1}\right|+\left|N_{1}\right|\right)/\left|PF^{*}\right|$$ (29)

In addition, the method takes into account both the number of solutions of the Pareto border approximation identified with each method and the required execution time.

#### Statistical tools

The proposed statistical analysis of the performance measures is a multivariate analysis of variance (MANOVA) [28]. This method verifies if, on average, any factor exerts a statistically significant effect on the mean vector of the response variables (one-way MANOVA). The hypothesis to be tested using the MANOVA is depicted in formulas (30) and (31):

$$H_{0}:{\vec{\mu }}_{1}={\vec{\mu }}_{2}=\ldots ={\vec{\mu }}_{m}$$ (30)

$$H_{1}:{\vec{\mu }}_{i}\neq {\vec{\mu }}_{j},\;\mathrm{for}\;\mathrm{some}i\neq j$$ (31)

Or more explicitly, the calculations are shown in formulas (32) and (33):

$$H_{0}:\left[\begin{array}{c} \mu_{11}\\ \ldots \\ \mu_{1l} \end{array}\right]=\left[\begin{array}{c} \mu_{21}\\ \ldots \\ \mu_{2l} \end{array}\right]=\cdots =\left[\begin{array}{c} \mu_{m1}\\ \ldots \\ \mu_{ml} \end{array}\right]$$ (32)

$$H_{1}:\left[\begin{array}{c} \mu_{i1}\\ \ldots \\ \mu_{il} \end{array}\right]\neq \left[\begin{array}{c} \mu_{j1}\\ \ldots \\ \mu_{jl} \end{array}\right],\mathrm{for}\;\mathrm{some}i\neq j$$ (33)

Where ${\vec{\mu }}_{i}$ is the vector of means of the response variables due to the *i*-th factor, while *μ*_*ij*_ is the average of the *j*-th response variable due to the effect of the ith factor. The general linear model is shown in Eq (34):

$${\vec{x}}_{ij}=\vec{\mu }+{\vec{\tau }}_{i}+{\vec{\varepsilon }}_{ij}$$ (34)

Where:

${\vec{x}}_{ij}$: Vector of response variables for the *i*-th level of the factor in the *j*-th observation.

$\vec{\mu }$: Population average vector, common to the factors.

${\vec{\tau }}_{i}$: Effect of factor *i* on the vector of response variables.

${\vec{\varepsilon }}_{ij}$: Random error of the response variable vector.

In this paper, the factors correspond to the different algorithm employed, i.e., Ɛ-constraint method, NSGA-II and SPEA2 methods, whereas the response variables are the two performance measures of *CPU Time* and *Extent*. The probability of a type I error was set to *α* = 0.05 (5.0%). Computations for the statistical analysis were performed using R programming on IDE RStudio and an Intel^®^ core i7-7500 quad core processor operating at 2.70 GHz. Moreover, a four hypothesis contrast test was conducted, namely, Hotelling-Lawley, Roy, Pillai and Wilk [29].

## Results

### Test instances

The six problem instances mentioned above were associated with children diagnosed with obesity in three age groups: 4–8 years, 9–13 years and 14–18 years. Each designed instance differs in the amount of recommended daily energy, suggested dietary fiber, and recommended amounts for some or all of the micronutrients considered (see Table 1). The notations in Table 1 that characterize the instances include three elements. The letter indicates the gender (“a”: girl; “o”: boy), the first number indicates the lower limit of the age range, and the second number indicates the upper limit of the age range, for example, o:4–8 indicates a boy aged 4–8 years.

**Table 1:** Test instances for each gender (a/o) and age group.

	o:4–8	a:4–8	o:9–13	a:9–13	o:14–18	a:14–18
Energy [kJ/day]	5857.6	5020.8	7531.2	6694.4	9204.8	7531.2
Vitamin A [μg/day]	400/1300	400/1300	600/1700	600/1700	900/2800	700/2800
Vitamin B1 [mg/day]	0.6/*ND*	0.6/*ND*	0.9/*ND*	0.9/*ND*	1.2/*ND*	1/*ND*
Vitamin B2 [mg/day]	0.6/*ND*	0.6/*ND*	0.9/*ND*	0.9/*ND*	1.3/*ND*	1/*ND*
Vitamin B3 [mg/day]	8/15	8/15	12/20	12/20	16/30	14/30
Vitamin B6 [mg/day]	0.6/40	0.6/40	1/60	1/60	1.3/80	1.2/80
Vitamin B9 [μg/day]	200/400	200/400	300/600	300/600	400/800	400/800
Vitamin B12 [μg/day]	1.2/*ND*	1.2/*ND*	1.8/*ND*	1.8/*ND*	2.4/*ND*	2.4/*ND*
Vitamin C [mg/day]	25/650	25/650	45/1200	45/1200	75/1800	65/1800
Vitamin E [mg/day]	7/140	7/140	11/220	11/220	15/260	15/260
Calcium [mg/day]	1000/2500	1000/2500	1300/3000	1300/3000	1300/3000	1300/3000
Copper [mg/day]	0.44/3	0.44/3	0.7/5	0.7/5	0.89/11	0.89/8
Iron [mg/day]	8/11	8/11	8/11	8/11	11/20	15/20
Magnesium [mg/day]	150/250	150/250	300/400	300/400	300/400	300/400
Phosphorus [mg/day]	500/3000	500/3000	1250/4000	1250/4000	1250/4000	1250/4000
Potassium [mg/day]	2457/4500	2106/4500	3159/4500	2808/4500	3510/4700	3510/4700
Selenium [μg/day]	15/40	15/40	15/40	15/40	40/55	40/55
Sodium [mg/day]	500/1400	500/1200	500/1800	500/1600	500/2000	500/1800
Zinc [mg/day]	5/12	5/12	8/23	8/23	9/24	11/24
Dietary fiber [g/day]	12/20	12/20	15/30	15/30	20/40	20/40

Table 2 indicates the parameters that do not depend on certain instances and are the valid nutritional recommendations for children aged 4–18 years. Thus, Table 2 includes recommendations for the proportion of energy contributed by each macronutrient, the proportion of energy contributed by each mealtime, the proportion of energy contributed by different fatty acids, and minimum/maximum allowed food portion. Table 2 also indicates the recommendations for daily and weekly food group consumption proposed by the INTA. Furthermore, a seven-day planning period was considered for all instances because the proposed model independently considers the weekly planning period.

**Table 2:** Recommendations for macronutrients, mealtimes, fatty acids, portion sizes, and food groups (daily and weekly).

Parameter	Value	Food groups	*RGDI*_*g*_/*RGDS*_*g*_	*RGSI*_*g*_/*RGSS*_*g*_
*PEMI*_1_/*PEMS*_1_	0.45/0.65	Vegetable	2/8	7/56
*PEMI*_2_/*PEMS*_2_	0.10/0.30	Fruit	2/8	7/56
*PEMI*_3_/*PEMS*_3_	0.20/0.35	Dairy products	2/8	7/56
*ECI*_1_/*ECS*_1_	0.1/0.15	Fish	0/1	1/3
*ECI*_2_/*ECS*_2_	0.05/0.1	Red meat	0/1	1/3
*ECI*_3_/*ECS*_3_	0.3/0.4	Poultry	0/1	1/3
*ECI*_4_/*ECS*_4_	0.05/0.1	Egg	0/1	1/3
*ECI*_5_/*ECS*_5_	0.1/0.15	Noodles	0/1	2/5
*ECI*_6_/*ECS*_6_	0.2/0.3	Rice	0/1	2/5
*RL*_1_	0.1	Potatoes	0/1	2/5
*RL*_2_	0.1	Legumes	0/1	1/3
*RL*_4_	0.1			
*RL*_5_	0.012			
*LIP*/*LSP*	0.5/1.5			

### Numerical results

The result of the performance measures are summarized in Table 3. Because the metaheuristics are stochastic processes, the average value of 10 executions is shown. For the Ɛ-constraint method, GAMS uses a Branch-and-Cut algorithm, and therefore only a single execution is performed. As an example of solutions obtained using these methods, Table 4 shows one of the Pareto border solutions for each method for one day for 4- to 8-year-old children. Importantly, the Pareto border is composed of a set of solutions that are not dominated by each other. The produced menus generally show that all methods repeat certain types of food, with a strong relationship between price and nutritional benefit (e.g., skim milk, natural yogurt, and lentils with rice). Notably, the evolutionary methods select many of the foods selected by the exact method.

**Table 3:** Computational performance of methods in groups stratified according to gender (a/o) and age.

Method	Indicators	o:4–8	a:4–8	o:9–13	a:9–13	o:14–18	a:14–18
Ɛ-constraint method (1)	No. of solutions	48	46	51	50	51	49
	*Extent*	54.69	52.48	51.09	52.69	61.73	59.20
	*CPU Time* [s]	57192.53	61397.81	55013.72	60227.17	55178.29	72538.67
	*Cont* (1/2)	0.21	0.2	0.19	0.2	0.17	0.18
	*Cont* (1/3)	0.37	0.4	0.33	0.33	0.29	0.28
NSGA-II (2)	No. of solutions	300	300	300	300	300	300
	*Extent*	39.57	36.25	44.09	45.66	50.75	50.67
	*CPU Time* [s]	123.76	133.90	149.18	127.86	124.97	122.95
	*Cont* (2/1)	0.79	0.8	0.81	0.8	0.83	0.82
	*Cont* (2/3)	0.58	0.73	0.65	0.64	0.65	0.62
	*Generational Distance*	36.57	52.27	30.18	35.50	34.34	22.59
SPEA2 (3)	No. of solutions	150	150	150	150	150	150
	*Extent*	39.49	32.62	46.98	46.13	51.53	50.32
	*CPU Time* [s]	274.42	283.83	277.41	280.41	266.06	268.41
	*Cont* (3/1)	0.63	0.6	0.67	0.67	0.71	0.72
	*Cont* (3/2)	0.42	0.27	0.35	0.36	0.35	0.38
	*Generational Distance*	44.30	59.38	23.03	22.22	18.03	19.83

**Table 4:** Menus created using the Ɛ-constraint, NSGA-II and SPEA2 methods.

Mealtime	Dish	Method					
		Ɛ-constraint		NSGA-II		SPEA2	
		Food	Portion	Food	Portion	Food	Portion
Breakfast	Hot beverage	Skim milk	0.5	Quaker oats with milk	1.2	Skim milk	0.6
	Sandwich	Whole wheat bread with ham	0.5	Whole wheat bread with honey	0.9	Whole wheat bread with margarine	0.5
Morning snack	Fruit	Strawberries	0.6	Cantaloupe	1.0	Cherries	0.6
Lunch	Salad	Lettuce	1.4	Broccoli	1.0	Celery salad	1.4
	Main course	Fish soup	0.5	Boiled potatoes with egg	0.5	Chickpeas	0.5
	Dessert	Natural yogurt	0.5	Plum	1.1	Blueberries	1.4
Afternoon snack	Fruit	Cherries	0.5	Raspberries	1.1	Cherries	0.6
Teatime	Hot beverage	Skim milk	0.5	Quaker oats with milk	0.6	Quaker oats with milk	0.8
	Sandwich	Whole wheat bread with ham	0.5	Whole wheat bread with ham	0.7	Whole wheat bread with cottage cheese	0.5
Dinner	Salad	Lettuce	1.0	Green beans	0.5	Celery salad	1.4
	Main course	Lentils with rice	0.5	White fish with potato	1.2	Potato, pumpkin, and beef stew	0.5
**Objective Values**							
Z1 [USD]		4.76		2.53		3.78	
Z2 [mg]		96.1		143.5		105.5	
Z3 [u]		60.8		72.2		70.2	
Z4 [%]		0		12		7	

### Statistical analysis

Given the inherent randomness of the solution methods employed, a thorough statistical analysis was conducted on the results of performance measures using the tools introduced in Section *2*.*3*.*3* to adequately compare the methods.

#### Statistical comparison of the performance measures *CPU time* and *Extent*

For a MANOVA to be appropriate, a non-negligible correlation must exist between the response variables. Thus, in this study, the correlation between the *CPU Time* and *Extent* was calculated, obtaining a Pearson’s linear correlation coefficient 𝑟 = 0.692746, indicating that the application of the MANOVA is appropriate.

The null hypothesis of MANOVA, namely, the factors do not exert a statistically significant effect on the mean vector of the response variables, was considered. The results of the various tests performed indicate that this null hypothesis must be rejected because the factors exert a statistically significant effect on the response variables. First, an a priori natural-log transformation was performed on the observations obtained using the models. Second, a four hypothesis contrast test was conducted, namely, Hotelling-Lawley, Roy, Pillai and Wilk [29]. Third, the assumptions of the MANOVA model were tested, i.e., multivariate normality, homogeneous covariance matrix and independence. Table 5 summarizes these results. Notably, p-values less than 5% for all the tests corroborate the findings mentioned above.

**Table 5:** Summary of assumptions and hypothesis testing using one-way MANOVA.

Item	Statistical Test	p-value
Hypothesis Test	Hotelling-Lawley	< 2.2e-16
	Roy	< 2.2e-16
	Pillai	0.0002204
	Wilks	< 2.2e-16
Assumption: Multivariate Normality	Skewness criteria	0.5053
	Kurtosis criteria	0.32
	Skewness and kurtosis criterion	0.3519464
Assumption: Homogeneous covariance matrix	Box’s M-test	0.07138
Assumption: Independence	Durbin-Watson	0.4583

**Fig 3:**
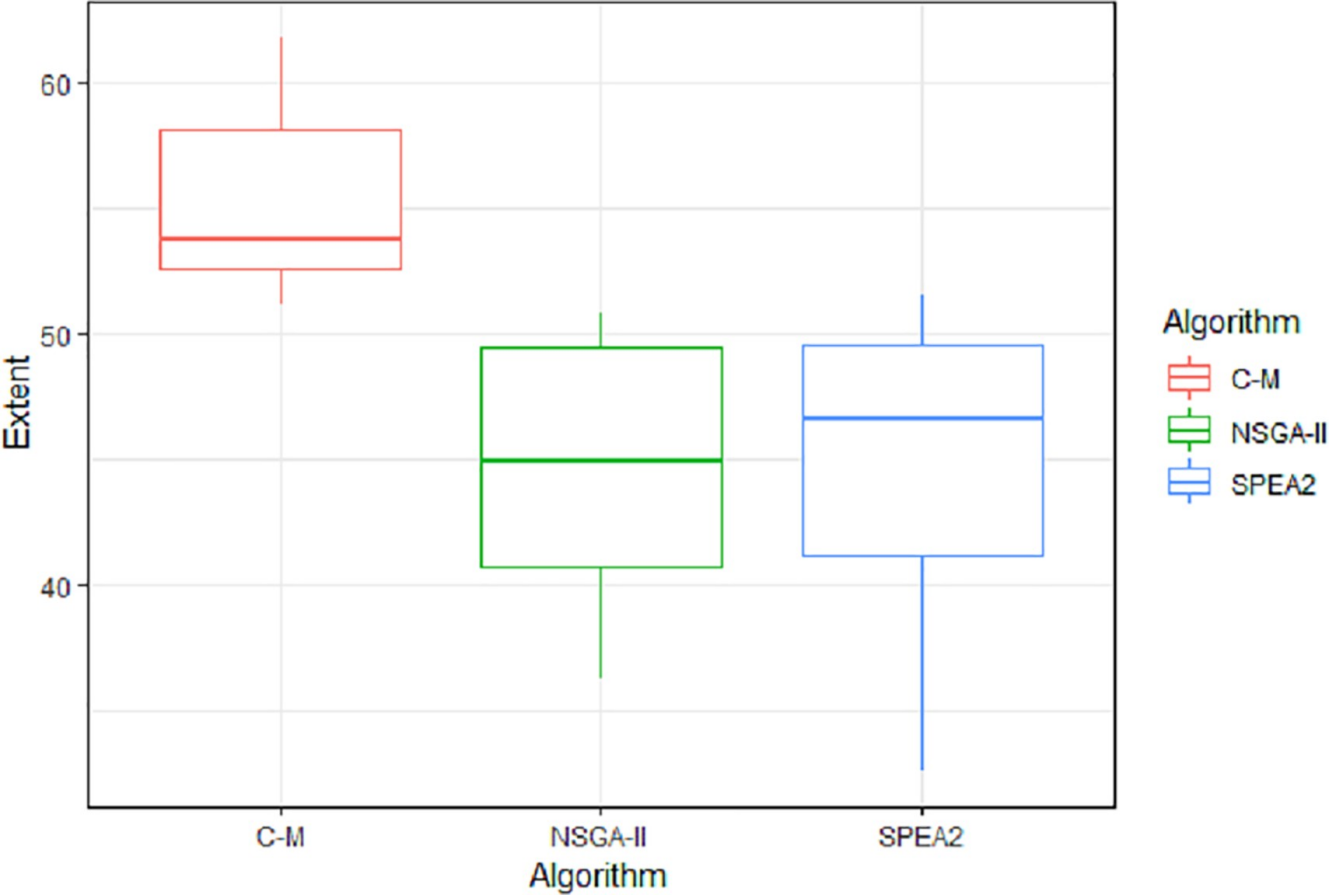
Box plot of the factors for the *Extent* variable.

**Fig 4:**
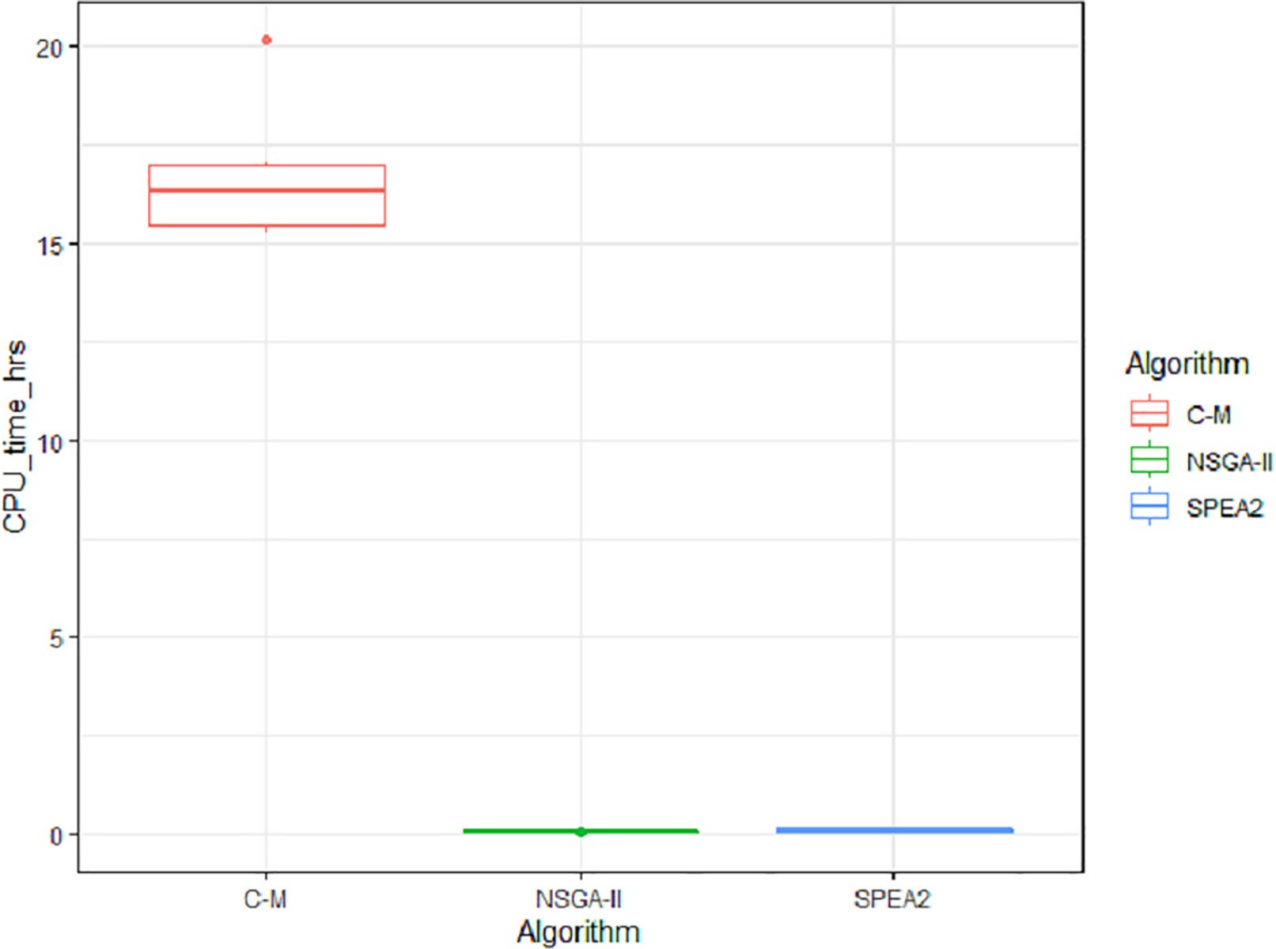
Box plot of the factors for the *CPU Time* variable.

The tests of differences in means between pairs of factors, the 95% confidence intervals (in terms of the original variables) and the p-values are shown in Table 6. Based on these results and the distribution of the values shown in Figs 3 and 4, we concluded that a significant difference in the *Extent* variable exists between NSGA-II & Ɛ-constraint and between SPEA2 & Ɛ-constraint, with the Ɛ-constraint method being superior to the NSGA-II & SPEA2 methods. On the other hand, the difference in *Extent* between the methods NSGAII & SPEA2 is not statistically significant. Regarding *CPU Time* in hours, we concluded that the methods NSGA-II & SPEA2 are superior to the Ɛ-constraint method, while the differences in the mean values calculated using the three methods is not statistically significant.

**Table 6:** Tests of differences in mean values between pairs of factors.

Variable	Compared factors	Average difference	Lower limit	Upper limit	p-value
*Extent*	NSGA-II & Ɛ-constraint	-10.815	-19.6341	-1.9958	0.0159
	SPEA2 & Ɛ-constraint	-10.802	-19.6208	-1.9825	0.0161
	SPEA2 & NSGA-II	0.0134	-8.8058	8.8325	0.9999
*CPU Time* [hrs]	NSGA-II & Ɛ-constraint	-16.7021	-18.2785	-15.1257	0.0000
	SPEA2 & Ɛ-constraint	-16.6619	-18.2383	-15.0855	0.0000
	SPEA2 & NSGA-II	0.0402	-1.5363	1.6166	0.9976

#### Statistical comparison of the performance measure *Generational Distance*

The proposed statistical analysis is an analysis of variance (ANOVA) [28], as we are interested in the effect of one factor (with two levels) on a unique response variable. The factor is, again, the different algorithms employed, i.e., NSGA-II and SPEA2, excluding the algorithm C-M (which is not a population-based algorithm). The response variable is the performance measure of *Generational Distance*.

Computations performed in this analysis were executed on the same machine as the one-way MANOVA, resulting in an acceptance of the null hypothesis with a p-value of 0.616, i.e., a statistically significant difference in the performance of *Generational Distance* is not observed. As usual, assumptions are held, and they are summarized in Table 7.

**Table 7:** Summary of assumptions for the one-way ANOVA.

Assumption	Statistic Test	p-value
Normality	Shapiro-Wilk	0.0818
Homoscedasticity	Bartlett	0.2584
Independency	Durbin-Watson	0.7651

## Discussion

The performance measures that were analyzed in this study are the *CPU Time*, *Extent* and *Generational Distance* (the latter was analyzed using the population-based metaheuristics). Based on the statistical analysis, the Ɛ-constraint method produced better results in terms of diversity than the metaheuristic methods, but at the expense of a significantly longer execution time than required by metaheuristics. A potential explanation for this finding is that the Ɛ-constraint method solves multiple versions of the same model with different combinations of epsilon (ε) values and the software (GAMS) uses the exact Branch-and-Cut method. Regarding the comparison between the metaheuristic methods used in the present study, no statistically significant differences are observed in the *Extent*, *CPU Time* and *Generational Distance* indicators.

On the other hand, *Cont* indicators were used to measure the proportion of individuals in a Pareto front approximation built by combining two populations. The contribution of the Ɛ-constraint method was less than 40%, although in all studied cases, the individuals included in the Pareto front approximation generated using this method still belonged to the approximation built from its combination with the population generated using the NSGA-II and SPEA2 methods. Interestingly, although all the solutions of the Ɛ-constraint method were part of the Pareto border built by combining their solutions with those obtained using metaheuristics, the metaheuristic methods provided a large percentage of solutions that were not dominated by the solutions obtained using the exact Ɛ-constraint method. Additionally, the computational time required was considerably shorter. Notably, the value of this measure is influenced by the population size defined for each method.

## Conclusions

The multiobjective approach proposed in this paper generates menus that minimize the consumption of substances that are particularly harmful to obese children. It also minimizes the nutritional mismatch and cost of planning to avoid limited access to healthy diets because of economic issues, while complying with the nutritional recommendations of specialized organizations. The proposed model for MO-NMPP-CHO with the created instances was solved with a deterministic method and two metaheuristic methods.

Although childhood obesity is a multifactorial problem, the formation of healthy eating habits at an early age creates benefits over the long term. Thus, the multiobjective mathematical programming model for planning nutritional menus described in this paper appears to be an appropriate method to minimize exposure to the major risk factors for the development of chronic diseases associated with childhood obesity, the total cost of nutritional planning, and nutritional mismatch.

Nevertheless, the numerical results indicate that solving this type of problem using exact methods is not appropriate to address real or complex instances because of their execution time. Positive results are obtained using evolutionary techniques that require appropriate computational times. Although these techniques only represent an approximate analysis, health professionals can provide guidance to create monthly and weekly plans in a fast and personalized manner, based on the requirements of each child.
